# Elevated risk of stillbirth in males: systematic review and meta-analysis of more than 30 million births

**DOI:** 10.1186/s12916-014-0220-4

**Published:** 2014-11-27

**Authors:** Debapriya Mondal, Tamara S Galloway, Trevor C Bailey, Fiona Mathews

**Affiliations:** Biosciences, College of Life and Environmental Sciences, Hatherly Laboratories, University of Exeter, Prince of Wales Road, Exeter, EX4 4PS UK; Current address: School of Environment and Life Sciences, University of Salford, Room 322, Peel Building, Salford, UK; Mathematics, College of Engineering, Harrison Building, Mathematical and Physical Sciences, University of Exeter, Exeter, EX4 4QF UK

**Keywords:** Gender medicine, Pregnancy, Birth, Fetal loss, Sex

## Abstract

**Background:**

Stillbirth rates have changed little over the last decade, and a high proportion of cases are unexplained. This meta-analysis examined whether there are inequalities in stillbirth risks according to sex.

**Methods:**

A systematic review of the literature was conducted, and data were obtained on more than 30 million birth outcomes reported in observational studies. The pooled relative risk of stillbirth was estimated using random-effects models.

**Results:**

The crude mean rate (stillbirths/1,000 total births) was 6.23 for males and 5.74 for females. The pooled relative risk was 1.10 (95% confidence interval (CI): 1.07–1.13). The attributable fraction in the whole population was 4.2% (95% CI: 3.70–4.63), and the attributable fraction among male fetuses was 7.8% (95% CI: 7.0–8.66). Study populations from countries with known sex-biased sex selection issues had anomalous stillbirth sex ratios and higher overall stillbirth risks than other countries, reflecting increased mortality among females.

**Conclusions:**

Risk of stillbirth in males is elevated by about 10%. The population-attributable risk is comparable to smoking and equates to approximately 100,000 stillbirths per year globally. The pattern is consistent across countries of varying incomes. Given current difficulties in reducing stillbirth rates, work to understand the causes of excess male risk is warranted. We recommend that stillbirths are routinely recorded by sex. This will also assist in exposing prenatal sex selection as elevated or equal risks of stillbirth in females would be readily apparent and could therefore be used to trigger investigation.

**Electronic supplementary material:**

The online version of this article (doi:10.1186/s12916-014-0220-4) contains supplementary material, which is available to authorized users.

## Background

Stillbirths are one of the most important, yet most poorly understood, adverse outcomes of pregnancy [1]. The definition of stillbirth varies between countries and over time, but generally refers to the death of a fetus in the later stages of pregnancy (cut-offs varying from 20 to 28 weeks gestation). Worldwide, around 2.6 million stillbirths occurred in 2009, according to the first comprehensive set of estimates [2,3]. This is similar to the number of early neonatal deaths, and is approximately half of the total child deaths (aged one to five years) occurring in the same period [4].

Stillbirth rates vary sharply by country, ranging from two per 1,000 total births in Finland to more than 40 per 1,000 total births in Nigeria and Pakistan [3]. There are calls for stillbirth to be included as a Millennium Development goal [2] since the majority of cases occur in low- and middle-income countries. Here, approximately 45% occur intra-partum, reflecting a lack of skilled birth attendants and emergency obstetric care, but many of the remainder are unexplained [1]. In high-income countries, access to skilled care during pregnancy and parturition, and better management of medical disorders present before or during pregnancy (including syphilis, malaria, diabetes, hypertension, and placental dysfunction disorders [1,5]) have helped to reduce stillbirth rates considerably since the 1940s. Nevertheless, one in 200 women reaching 22 weeks gestation will have a stillborn baby [6]. For example, in the UK, which has one of the highest rates among high-income nations, stillbirths affect four times as many babies as Down’s syndrome. Globally, rates of stillbirth have declined only marginally over the last 15 years (estimated 1.1% between 1995 and 2009), and high-income, as well as low-income countries, follow this trend [3]. A better understanding of the aetiology of stillbirths is therefore crucial if further improvements are to be made.

Maternal factors including obesity, smoking, advancing maternal age, and low educational attainment have all previously been shown to be linked with stillbirth [6,7]. In this study, we investigated the impact of fetal sex. It is known that a range of adverse pregnancy outcomes are more common in males than females, and there are sex-specific differences in the growth and function of male and female placentae [8–10]. However, fetal sex is rarely considered explicitly as a risk factor for stillbirth, although it is sometimes recorded as a covariate [11]. For example, a recent special issue on stillbirth in *The Lancet* did not refer at all to sex; and in marked contrast to post-natal morbidity and mortality events, routine statistics in most countries do not even report stillbirths separately by sex. This issue is important for two reasons. First, as with other health outcomes, if sex imbalances exist, then explorations of the aetiology are warranted. Second, establishing normal patterns of risk in male and female fetuses can help identify situations where interventions against females may be occurring. We have therefore performed a meta-analysis of published studies, together with a large publicly accessible dataset from the UK.

## Methods

### Data sources

We searched ISI Web of Science and Medline for studies providing sex-specific rates of stillbirth and published between 1990 and 2012 inclusive. The techniques specified by Stroup et al. [12] for conducting and reporting meta-analysis of observational studies in epidemiology (MOOSE) were followed Additional file 1. The medical subject headings (MeSH) and search terms used were: “stillbirth”, “still-birth”, “fetal death”, “foetal death”, “pregnancy loss”, “sex”, “fetal sex”, “foetal sex”, and “gender”. The terms were combined with the Boolean operators “OR” or “AND”. The reference lists and cross-references of eligible studies were also searched, as were the bibliographies of recent reviews. Google and Google Scholar were used to search the ‘grey’ literature (information outside scientific journals) for relevant information. Studies not published in English and randomised trials testing interventions to reduce stillbirth and/or perinatal mortality were excluded. We first checked the abstracts of the studies identified on the basis of having relevant titles. The full text of studies regarded as potentially eligible was then assessed to determine whether they had provided data, stratified by sex, on both the numbers of stillbirths and the numbers of live births/total births. If the relevant numbers were not directly provided, the paper was included if the data could be extracted, using standard methodology, from other information provided (e.g., where un-adjusted odds ratios and total numbers of births were provided). The methodological quality of the studies was also assessed: those with incomplete definitions of, or missing data on, the population, study design, exposure, or outcome variables were excluded. Two reviewers (DM and FM) independently reviewed the methodological quality of the studies and differences were resolved by consensus and discussion.

The definition of stillbirth has changed over time, and there are also differences between countries despite the availability of international guidelines [13]. The research therefore included studies which used a variety of definitions of stillbirth, for example ≥28 weeks’ gestation or birth weight >500 g; or ≥20 weeks’ gestation or birth weight >400 g. Table 1 provides a summary of the studies and the definitions used by each.

**Table 1:** **Study characteristics**

**First author**	**Location, year [reference]**	**Study population and design**	**Stillbirth definition**	**Inclusion criteria**
Rasmussen	Norway, 1967–1998 [14]	Population-based retrospective cohort study	Unexplained antepartum fetal death defined as death before labour without known fetal, placental, or maternal pathology	All singleton births with at least 28 weeks of completed gestation
Gadow	Argentina, Bolivia, Brazil, Chile, Colombia, Costa Rica, Ecuador, Paraguay, Peru, Uruguay, and Venezuela, 1982–1986 [15]	Hospital-based retrospective cohort study	Death at ≥20 weeks of gestation and weight ≥500 g	All births, live or dead, weighing 500 g or more occurring in 102 participating hospitals distributed in 11 countries
Xu	Northern Finland, 1996	Population-based retrospective birth cohort study	Death at >28 weeks of gestation	All singleton births with at least 28 weeks of completed gestation and a birth weight of at least 1,000 g
	Northern Finland, 1985–86			
	Qingdao, China, 1992 [16]			
Smith	Scotland,1980–1996 [17]	Population-based retrospective cohort study	Death at ≥28 weeks of gestation and weight >500 g	All singleton first births weighing more than 500 g delivered between 28 and 43 weeks gestation in Scotland in 1980–1996
Petridou	Greece, 1989–1991 [18]	Population-based case-control study	Death at ≥28 weeks of gestation	All reported stillbirths within the National Statistical Service of Greece database between the study years with gestational age greater than or equal to 28 weeks
Kesmodel	Aarhus, Denmark, 1989–1996 [19]	Hospital-based retrospective cohort study	Death at ≥28 weeks of gestation	Danish women with singleton pregnancies who did not have an induced abortion and who provided information on alcohol intake while receiving routine antenatal care in the Department of Obstetrics and Gynaecology at Aarhus University Hospital, Aarhus, Denmark, between September 1989 and August 1996
Efkarpidis	Nottingham, UK, 1991–1997 [20]	Hospital-based case-control study	Fetal deaths diagnosed by ultrasound at ≥24 weeks of gestation	All singleton stillbirths excluding any <24 weeks gestation, at the Nottingham City Hospital between the study period formed the cases which were compared to a control group of pregnancies (n =499) delivered during the same time period, from same geographic population, selected using random allocation by computer
Nielsen	Tamil Nadu, India, 1995 [21]	Community-based prospective observational study	Not defined	All births within six months from the day of survey to Tamil speaking mothers residing in the survey area for more than two days and were not mentally retarded
Aliyu	Missouri, USA, 1, 1989–2005 [22]	Population-based retrospective cohort study	Death at ≥20 weeks gestation	Singleton births to mothers diagnosed with placental abruption within gestational age range of 20 to 42 weeks
Aliyu	Missouri, USA, 2, 1989–2005 [23]	Population-based retrospective cohort study	Death at ≥20 weeks gestation	Singleton births to mothers diagnosed with preeclampsia or eclampsia within the gestational age range of 20 to 42 weeks.
Engel	Newcastle, Australia, 1995–1999 [24]	Hospital-based retrospective cohort study	Not clearly defined. Based on the plot and results within the manuscript, stillbirths considered as death >20 weeks of gestation	All cases of singleton pregnancies for women aged 13 to 47 years at obstetric unit of John Hunter Hospital
Wen	Qingyuan, China, 1997–1998 [25]	Hospital-based retrospective study	Intrapartum fetal deaths at ≥20 weeks gestation or ≥500 g	All hospital-born (participating hospitals (n =18)) registered births between the study period
Sutan	Scotland, 1994–2003 [26]	Population-based retrospective cohort study	Unexplained antepartum stillbirth defined as deaths occurring before labour with no evident fetal, maternal, or placental abnormality sufficient to be considered as the cause of death	All singleton, pregnancies occurring at 20 completed weeks of gestation and more or occurring after the fetus reached a body mass of 200 g or more
Ingemarsson	Sweden, 1999–2000 [27]	Population-based retrospective study	Death at ≥28 weeks gestation	All pregnancies registered in the national medical birth registry with a gestational duration of at least 28 completed weeks or less if the infant was alive at birth
Mohsin	New South Wales, Australia, 1998–2002 [28]	Population-based retrospective cohort study	Death at ≥20 weeks and weight ≥400 g	All live births and stillbirths with at least 20 weeks gestation or with a birth weight of 400 g or more
Hadar	Petach Tikva, Israel, 1995–2007 [11]	Hospital-based retrospective cohort study	Death at >20 weeks of gestation or death when weight >500 g if gestational age unknown	All cases of stillbirths and overall deliveries during the study period
MacDorman	USA, 2003 [29]	Population-based retrospective study	Death at ≥20 weeks of gestation	All births in the year 2003 with 20 weeks of gestation or more
Yoonhee	Ghana, 2003–2008 [30]	Population-based cohort study	Death at ≥28 weeks of gestation	All pregnancies from 1^st^ July 2003 to 30^th^ September 2008 in seven contiguous rural districts
MacDorman	USA, 2004 [31]	Population-based retrospective study	Death at ≥20 weeks of gestation	All births in the year 2004 with 20 weeks of gestation or more
MacDorman	USA, 2005 [32]	Population-based retrospective study	Death at ≥20 weeks of gestation	All births in the year 2005 with 20 weeks of gestation or more
Mutihir	Nigeria, 2006–2007 [33]	Hospital-based prospective observational study	Death at ≥28 weeks of gestation	All births delivered at the maternity unit of Jos University teaching Hospital between Jan 2006 and April 2007
National Statistics (Office of)	England and Wales, 1990–2010	Population-based retrospective cohort study	Death at >24 weeks of gestation (or prior to 1993, death at >28 weeks of gestation)	Summary data on live and stillbirths by each year, published by The Office of National Statistics, UK

### Inclusion and exclusion criteria

Data were included in the meta-analysis if they came from studies that fulfilled all of the following criteria:

- Original observational epidemiological study (including community-based cross-sectional studies; case-control studies and cohort studies)
- Reported stillbirth rates
- Reported either raw numbers of stillbirths and live births by sex, or provided unadjusted odds rations or relative risks (RRs) and total population size to permit relevant data to be abstracted into 2 × 2 tables
- Reported the definition used for stillbirth
- Described the study population
- Described the study design
- Reported the results in English

Randomised controlled trials and other assessments of interventions designed to reduce stillbirth or neonatal death rates were excluded.

### Office of National Statistics Vital Statistics data

The national data archive of population Vital Statistics in England and Wales was used to derive information on the numbers of live births and stillbirths by sex of the fetus for the years 1990 to 2010 (see [34]; theme population). This archive records all registered live and stillbirths.

### Data abstraction

After reading each relevant article that appeared to meet the inclusion criteria, data on pregnancy outcome by sex was abstracted into 2 × 2 tables. In addition, the following data were noted for each study: year of study, publication, sample method, sample size, study design (cohort, case-control, population-based cross-sectional summary reports of vital statistics), definition of stillbirth, and any adjustments made for potential confounding variables in the original article, the adjusted RRs with confidence interval (CI), and the variables included in the final models. The data abstraction was done by PM and checked by FM. Decisions on whether to exclude studies, all of which were based on the absence of necessary data defined in the inclusion criteria, were reached following discussion between the authors.

### Statistical analysis

RRs were used to quantify the relationship between sex and the prevalence of stillbirth. We combined the data using DerSimonian and Laird random effects (inverse variance) models to estimate the pooled RRs and associated 95% CIs. These models are preferred because we suspected *a priori* that differences between populations (particularly low-/medium- vs. high-income countries) and study designs could be important [35]. The random effects model assumes that all studies are estimating different effects resulting from variations in factors, such as study population, and samples variation within and between studies. As a result, it generally produces wider confidence intervals than fixed effect models, the pooled results of which we also present for comparative purposes. Population-attributable risk estimating the percentage of the stillbirth in the population due to male sex was calculated [36].

Funnel plots were assessed for symmetry to evaluate possible publication bias. Statistical heterogeneity across the studies was explored through subgroup analyses of the following study-level covariates on the RRs associated with sex: study setting, study population, and study design. These assessments were supplemented with the inspection of I^2^ statistics which estimates the percentage of outcome variability that can be attributed to heterogeneity across studies [37]. An I^2^ value of 0% denotes no observed heterogeneity, whereas 25% is “low”, 50% is “moderate”, and 75% is “high” heterogeneity. Finally, sensitivity analyses were performed to explore the effects of heterogeneity between study types. The potential influence of each individual study on the overall summary estimates was assessed by re-running the meta-analysis omitting one study at a time. Analyses based on rates adjusted for covariates increase the likelihood that any associations represent independent relationships between the exposure variable (fetal sex) and outcome (stillbirth). We therefore assessed if the effect sizes would have been materially altered by using adjusted rather than crude data, and provide the pooled adjusted RRs for comparative purposes where the data permit.

The analyses were performed using statistical software R-version 2.15.2 [38]. Meta analyses were conducted with the package Meta-version 2.0-2 [39], and attributable fractions were calculated using EpiR version 0.9-48 [40].

## Results

### Literature search

**Figure 1:**
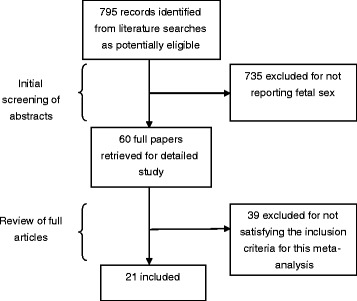
**Flow diagram of study selection.**

The literature search identified 795 unique citations. After detailed review, 21 of these studies met the inclusion criteria (Figure 1).

### Study characteristics

The characteristics of the studies are summarized in Table 1. All the published studies reported data from a single study population, with the exception of Xu et al. [16], who reported data from three different study populations, and Gadow et al. [15], who reported data from 11 Latin American countries. Each of these countries was treated as a separate population in the analysis. With the original dataset from England and Wales added, 34 populations were available for the meta-analysis. Only two of the populations were studied using a case-control design. Given that stillbirth is a relatively rare outcome, we interpret the various estimates of risk (i.e., odds ratio, RR) as being approximately equivalent, and here report RRs.

**Figure 2:**
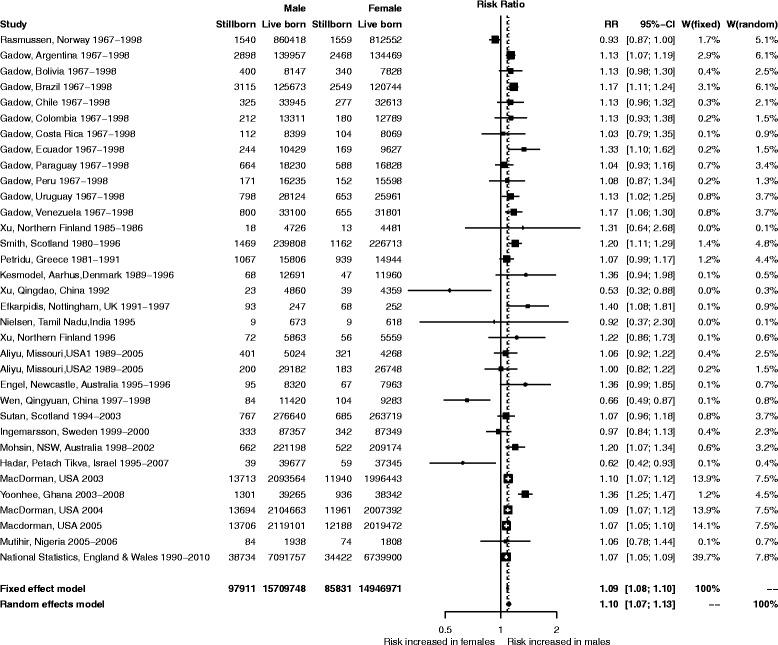
**Forest plot showing relative risk of stillbirth associated with male sex based on weights of individual studies.**

The risk of stillbirth was greater for male than female fetuses (Figure 2): the crude mean rate (stillbirths/1,000 total births) was 6.19 for males and 5.71 for females in the study cohorts. There was significant heterogeneity among studies (I^2^ = 71.9% (95 CI: 60.4%–80.0%)), but the pooled risk estimates were similar whether we used a random effects model (RR =1.10, 95% CI: 1.07–1.13) or a fixed effects model (RR =1.09, 95% CI: 1.08–1.10). The attributable fraction due to sex in the population was 4.17% (95% CI: 3.70–4.63), and the attributable fraction among male fetuses was 7.82% (95% CI: 6.97–8.66).

### Publication bias

**Figure 3:**
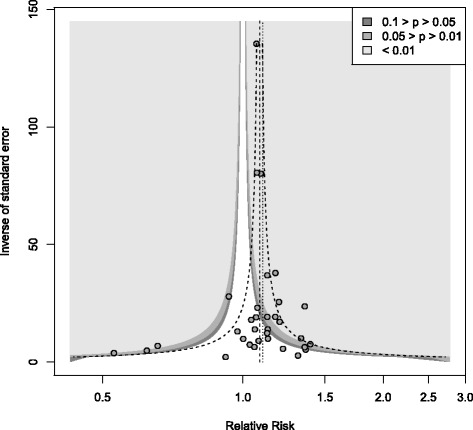
**Contour-enhanced funnel plots for the meta-analysis (random effects model).**

The contour-enhanced funnel plot for this meta-analysis is shown in Figure 3. There is some asymmetry indicating the possibility of missing studies. However, the asymmetry is not marked, and inspection of the graph indicates that the apparently missing studies largely fall in the area of statistical significance (shaded area) rather than non-significance (white area). True heterogeneity, rather than a failure to publish non-significant results, is therefore the most plausible explanation of the observed patterns.

### Subgroup analyses

**Figure 4:**
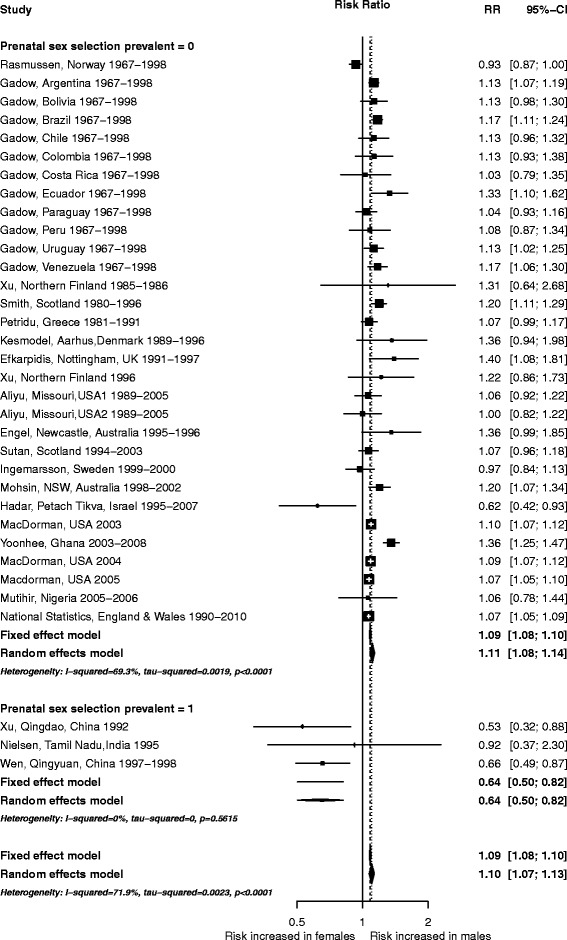
**Subgroup meta-analysis separating countries with known issues of prenatal sex selection and remaining countries.**

There was no evidence for a difference in RRs according to the income classification of the country (Q =0.37, df =1, *P* =0.543, Additional file 2). Since prenatal sex selection could bias the data on sex-specific stillbirth rates, we conducted subgroup analyses of countries identified by the World Health Organisation [41] as having biased prenatal sex selection (n =3) and those that do not (n =31) (Figure 4). This reduced the observed heterogeneity. For the former, the overall risk of stillbirth was higher (mean rate of 8.5 stillbirths per 1,000 births in affected countries compared with 6.0 elsewhere), and females had a higher relative risk (RR =0.64, 95% CI: 0.50–0.82). This switch in the direction of sex-linked risk appears to be based on higher than expected death rates for females rather than reduced fatalities in males. Given the live-birth rates in the study cohorts from India and China, if stillbirth patterns had followed those seen in other countries we would expect approximately 186 cases, 89 of them female (95% CI: 87.0–91.5) (based on the random effects model), whereas 116 male and 152 female stillbirths were actually recorded. There were only two studies from countries without known significant prenatal sex selection, where the risk of stillbirth was higher in females than males [11,14]. In each case, overall stillbirth rates were unusually low, suggesting that prenatal sex selection is unlikely to be responsible for the observed patterns.

### Sensitivity and influence analyses

For the countries with no known sex-biased selection, the overall heterogeneity estimate (I^2^) was 69.3% (95% CI: 55.7%–78.8%). We therefore conducted sensitivity analyses, based on random-effects models, to explore the reasons for the remaining heterogeneity (Additional file 3). First, we explored the effect of the definition of stillbirth, by grouping studies according to the gestational age cut-off. Two thirds (20/31) of the studies used a definition that included relatively early gestational ages (≥20 weeks). Within this subgroup, heterogeneity was reduced (I^2^ = 39.8%: 95% CI: 0%–64.7%) and the stillbirth RR for males remained similar to the pooled estimate derived from all studies. Where a gestational age cut-off ≥28 weeks was used, the RR of stillbirth remained similar (RR =1.12, 95% CI: 1.00–1.26 compared with RR =1.11, 95% CI: 1.08–1.13), but heterogeneity remained high (I^2^ = 85.4%, 95% CI: 74.2–91.8). Hence, for gestational age ≥28 weeks, we explored the effect of population type (hospital- versus population-based) and study design (case-control vs. cohort/population cross sectional survey). In all subgroups, the risk was elevated for male fetuses, and there was no difference in the effect size between the subgroups. The subgrouping had no material effect on heterogeneity (Additional file 3).

We explored whether the findings were consistent for the studies (n =8) which reported adjusted RRs (Additional file 4) (note that covariates varied widely between the studies). The pooled adjusted RRs had similar effect size as the overall result, but heterogeneity was lower (Additional file 3).

**Figure 5:**
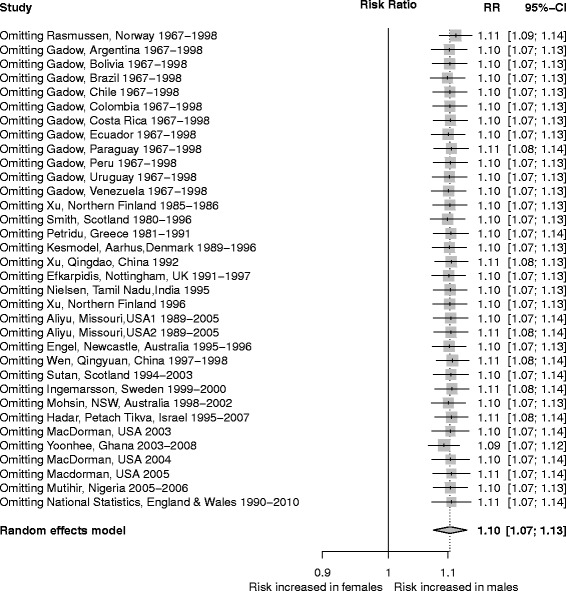
**Sensitivity analysis showing effect of leaving out each study in turn on overall estimate of RR.**

Sensitivity analyses which left out individual studies in turn found no evidence that any study exerted particular influence on the pooled RR estimate (Figure 5). This suggests that findings of the meta-analysis are robust.

## Discussion

This meta-analysis, which includes data on more than 30 million births, links sex with stillbirth, the risk being about 10% higher in male fetuses. We estimate that about 4% of stillbirths in the whole population are sex-associated and, among male fetuses, 7.8% of cases are attributable to sex. This is comparable to the excess risk due to smoking and a little less than that for primiparity [6]. Globally, this disproportion equates to approximately 100,000 stillbirths per year.

A highly consistent pattern of excess male mortality was seen across different populations and income groups. However, three studies from China and India, countries where sex-biased induced abortion is a known issue [15], showed reversed trends [16,21,25]. Given that there was no evidence of any reduction in male risk of stillbirth in the Chinese or Indian studies, and overall stillbirth rates were higher than expected, the most plausible explanation of the data is late intervention against female fetuses as a means of prenatal sex selection.

Different risk factors might be expected to operate at different gestational ages. When stillbirth is defined using an early cut-off point, such as 20 weeks gestation, cases are likely to include late spontaneous abortions caused by malformations, chromosomal abnormalities, and congenital infections [42]. In contrast, where the cut-off is placed late (≥28 weeks), the group is more restricted, and includes a greater proportion of cases with an apparently normal pregnancy in which no specific problems are documented and intra-partum fetal deaths [14]. However, we found no evidence that the risks to males differed according to whether the cut-off was placed at 20 weeks gestation (RR =1.11, 95% CI: 1.08–1.13) or at 28 weeks (RR =1.12, 95% CI: 1.00–1.26).

The study of stillbirth is complicated by the large sample sizes required to study a relatively rare outcome, and by the varied causes that may contribute in different populations [24]. Substantial agreement between fixed and random effects models increases confidence in the results, and reflects the fact that the studies are large and include data on more than 180,000 cases [43].

The observed heterogeneity (I^2^ = 64.6%) is reasonable given the range of study designs and definitions of stillbirth with respect to gestational age. There is little evidence of publication bias, with the slight asymmetry in the funnel plot being more likely to reflect the heterogeneity between studies. It was not possible to distinguish ante-partum from intra-partum stillbirths in most published studies, despite the obvious differences in aetiology and potential for intervention. Given that there was no evidence of any study biasing the results, we conclude that the meta-analysis is robust. Indeed our results may be conservative as the meta-analysis is based on crude rates and not on adjusted rates for potential confounders which gave slightly higher estimates. We used unadjusted data because most studies did not report adjusted rates separately for each sex. However, where appropriate data were available, the general pattern of increased risk for male fetuses was confirmed. Given the consistency of the observed effects across most nations, including those with highly-developed recording systems and medical care, it is unlikely that our results are artefacts of under-recording or misdiagnosis.

This meta-analysis identifies fetal sex as an important risk factor for stillbirth, and the approach has been useful in resolving apparent conflicts between publications [44]. It is known that differences in male and female development begin very early in life. For example, Y chromosome-linked genes are transcribed at the two-cell stage and, in animal models, male embryos have faster development and higher metabolic rates than females [45,46], potentially leaving the male fetus more vulnerable to a range of stressors, including endocrine fluctuations, oxidative stress, and nutritional compromise. Recent experimental work in animal models has demonstrated that gene expression in the murine placenta is adaptive and shaped by diet, with placental growth in males being more susceptible to nutritional compromise than that of females [47]. It is already known that risks of preterm delivery are greater in male than female infants [48] and sex-specific differences in placental structure and function among pregnancies complicated by preterm delivery have been demonstrated [49,50]. We have now confirmed that stillbirths are also higher in male fetuses.

## Conclusions

Given the population-attributable risk for stillbirth due to sex, understanding why males are at higher risk is a research priority that could potentially lead to sex-specific approaches to the management of high-risk pregnancies. The routine recording of stillbirth type by fetal sex would help uncover which types of stillbirth are sex-linked. In countries showing reversed patterns of stillbirth risk, work is warranted to clarify whether female feticide or other explanations can account for the elevated risks to females.

## Additional files

Additional file 1:
**Protocol for systematic review on fetal sex and risk of stillbirth.**
Additional file 2:
**Subgroup meta-analysis separating studies according to income classification of country.** Income group 1 = high; Income Group 2 = low/medium. Additional file 3:
**Summary of subgroup analyses.** Indented rows are subgroups of the higher categories. Additional file 4:
**Subgroup meta-analysis comparing results obtained from raw relative risks and those adjusted for covariates.**
