# Supplementary material for: Elevated risk of stillbirth in males: systematic review and meta-analysis of more than 30 million births
Source: BMC Med. 2014 Nov 27;12:220. doi: 10.1186/s12916-014-0220-4 (PMC4245790; doi:10.1186/s12916-014-0220-4)
Supplement: Additional file 1: — Protocol for systematic review on fetal sex and risk of stillbirth. [file 12916_2014_220_MOESM1_ESM.docx]

PROTOCOL FOR SYSTEMATIC REVIEW ON FETAL SEX AND RISK OF STILLBIRTH

| Main title | Fetal sex and risk of stillbirth: systematic review |
| --- | --- |
| Review group | The review team is comprised of three co-investigators and a research associate  Fiona Mathews (FM) is the Principal Investigator, is Senior Lecturer in the College of Life and Environmental Sciences at the University of Exeter. She conducts epidemiological research on the effects of environmental exposures on reproductive processes.  Tamara Galloway (TG)is Professor of Ecotoxicology in the College of Life and Environmental Sciences at the University of Exeter. She investigates the effects of environmental exposures on human and animal health.  Trevor Bailey (TB) is Professor of Statistics in the College of Engineering, Computing and Mathematics at the University of Exeter. His interests are in applied statistical modelling and spatial and environmental epidemiology.  Debapriya Mondal (DM) is the research associate with PHD in Health Risk Assessment.  DM and FM are responsible for acquiring the data, reviewing the published studies and performing the analyses. TG and TB will be responsible for providing guidance on analytical approaches and for reviewing and revising the systematic review. |
| Institutional base | University of Exeter |
| Authors (Contact details) | Dr Fiona Mathews (Principal Investigator)  Senior Lecturer in Mammalian Biology  & Programme Director for BSc. in Biology and Animal Behaviour  University of Exeter  Hatherly Laboratories  Prince of Wales Road  Exeter EX4 4PS  tel 0(44) 1392 263406 mobile 07977 137421  email f.mathews@exeter.ac.uk  Professor Tamara S Galloway (Co-investigator)  College of Life and Environmental Sciences  Geoffrey Pope Building  Stocker Road  Exeter EX4 4QD  tel 0(44) 1392 263436 mobile 0771 778 2774  fax 0(44) 1392 263700  email t.s.galloway@exeter.ac.uk  Professor Trevor Bailey  Associate Dean for Education  College of Engineering, Mathematical and Physical Sciences  University of Exeter  EX4 4QS, UK  tel 0(44) 1392 725223  email: t.c.bailey@exeter.ac.uk  Dr Debapriya Mondal(Research Associate)  Associate Research Fellow  College of Life & Environmental Sciences  University of Exeter  Hatherly Laboratories  Exeter EX4 4PS  Mobile-07952314201  email d.mondal@exeter.ac.uk |
| Registry number for systematic review | N/A |
| Conflict of interest | None |
| Acknowledgements | The project is funded by The Wellcome Trust |

**Background**

***Aim and rational for project***

As adults, males have higher morbidity and premature mortality than females. They also appear at greater at risk during intrauterine and neonatal life. Our objective in this Wellcome-Trust funded research project is to quantify the degree of excess risk to males across several linked outcomes in early life. By undertaking a cohesive examination of existing literature and databases, the work will identify the extent of gender imbalances and examine whether these are are increasing or static, and whether patterns are consistent across countries.

***Objective of review of stillbirth***

The overall objective is to perform a systematic review of published studies to investigate the impact of male gender on stillbirth, and to summarise the effect size using meta-analysis.

**Method**

***Identification of potential studies: Search strategy***

1. The search will be run in Medline on the Ovid platform and Web of Science on the Thomson Gale platform
2. Only published literature in English from 1990 will be considered
3. The search terms will be

Stillbirth/

Fetal death/

Foetal death/

Pregnancy loss/ and

sex/

fetal sex/

foetal sex/

gender

4. Citations identified from electronic searches will be downloaded to a Reference Manager database. Abstracts will be screened for relevance and full copies of studies that may meet the inclusion criteria will be obtained. Reference lists of relevant systematic reviews and of included studies will also be screened for potentially relevant primary studies. Authors will be contacted for further information or relevant unpublished data where necessary.

***Inclusion and exclusion criteria***

1. Type of measure

Inclusion:

1. Studies providing numbers of stillbirths and the numbers of live births or total births stratified by sex will be included
2. Studies providing information such as un-adjusted odds ratios from which the relevant numbers of stillbirths and the numbers of live births can be estimated for both males and females will be included

Exclusion:

1. Studies from which a cross-tabulation of numbers of live-births and stillbirths by gender will be excluded

2. Method

Inclusion:

1. Original epidemiological observational studies (including Case-Control or Cohort studies, and cross-sectional reports of populations) with described study design will be included.

Exclusion:

1. Those with incomplete definitions of, or missing data on the population or study design will be excluded.
2. Studies with missing or incomplete, definitions of exposure (gender) or outcome (live- or stillbirth) will be excluded.
3. Randomised controlled trials of interventions targeting stillbirth and neonatal mortality

***Screening studies***

Inclusion and exclusion criteria will be applied first to abstracts and then to full manuscripts. Full manuscripts will be obtained for those studies where the abstract appears to meet the criteria or where there is insufficient information to be certain about excluding them. The inclusion and exclusion criteria will be re-applied to the full manuscripts and those that do not meet the criteria will be excluded.

***Characterising the studies***

The included studies will be characterised based on year of study, population type, study design and definition used for stillbirth. A data extraction form will be developed and the data extracted relevant to study characteristics will be summarised. The form will be piloted by DM and FM and any necessary amendments will be discussed to make modifications to the form if required.

***Quality assurance process***

Data will be extracted by DM and checked by FM. Any disagreements will be resolved by discussion, and if necessary by discussing with others in the team. Where data are not available in the published report, the authors will be contacted for the missing information. Where relevant data is not available or the author not contactable, then the data will be assessed qualitatively.

Methodological quality of included studies will be assessed considering study design, selection bias, confounders, data collection methods, withdrawals and dropouts, and integrity of the study method. Two reviewers (DM and FM) will assess quality of studies independently. Any disagreements in quality ratings will be resolved by discussion.

***Process of synthesis***

All studies will be included in the synthesis, according to the inclusion and exclusion criteria identified in previous sections. The outcome data will be stillbirths and live-births stratified by sex into a 2 x 2 table for each study.

The primary analyses will examine the output using meta-analysis methods (fixed and random effect models; the choice of appropriate model being influenced by the heterogeneity of the studies) and appropriate graphical methods (forest plots).We will explore heterogeneity using quantitative measure (I-square statistics) and graphical measures (funnel plot) and confounding effects will be looked by performing secondary analyses using subgroup or sensitivity analysis

***Deriving conclusions***

We will use a participatory means of drawing inferences and conclusions from our results. In order to do so, preliminary findings will be synthesised and integrated as a report by DM and FM and circulated to TG and TB. The final conclusions will result from debate and discussion within the team. We will then share the findings as a manuscript publish in a reputed journal
