# Supplementary figures and images for: Elevated risk of stillbirth in males: systematic review and meta-analysis of more than 30 million births
Source: BMC Med. 2014 Nov 27;12:220. doi: 10.1186/s12916-014-0220-4 (PMC4245790; doi:10.1186/s12916-014-0220-4)

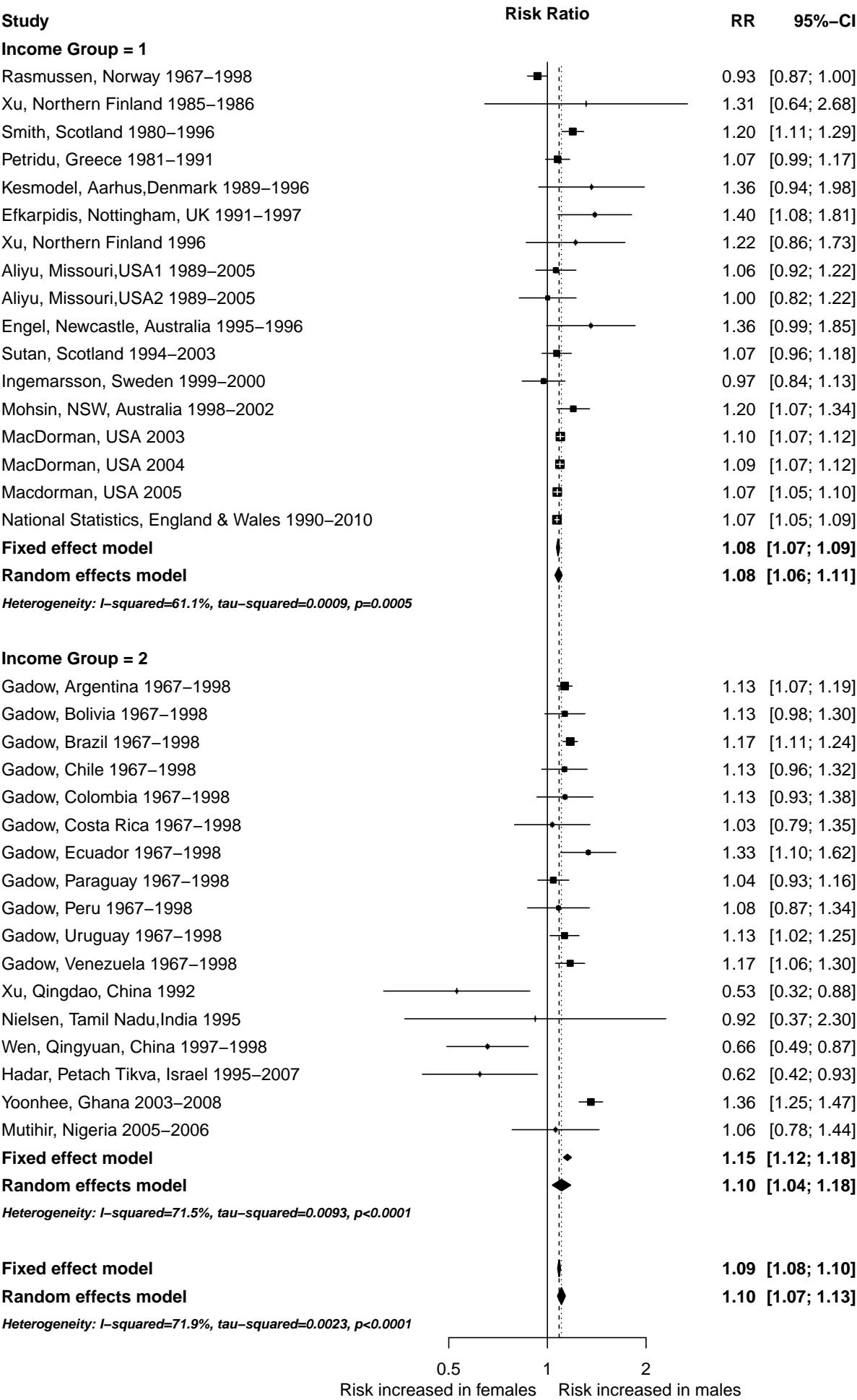

Supplement: Additional file 2: — Subgroup meta-analysis separating studies according to income classification of country. Income group 1 = high; Income Group 2 = low/medium. [file 12916_2014_220_MOESM2_ESM.pdf]

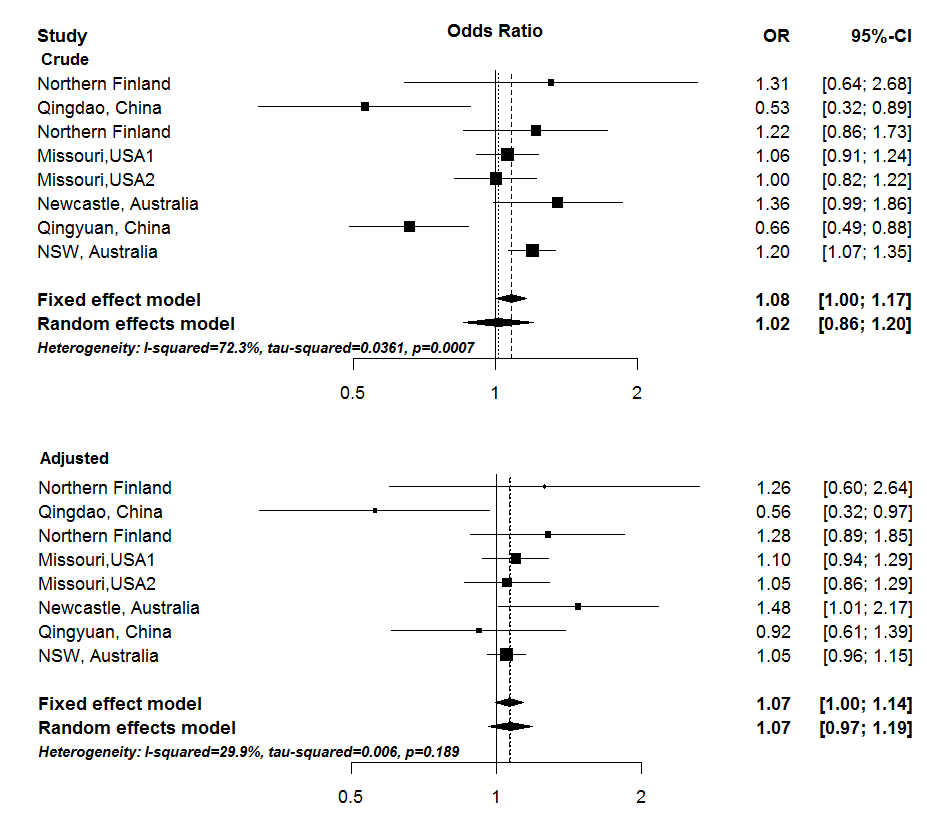

Supplement: Additional file 4: — Subgroup meta-analysis comparing results obtained from raw relative risks and those adjusted for covariates. [file 12916_2014_220_MOESM4_ESM.tiff]
