# Supplementary material for: Elevated risk of stillbirth in males: systematic review and meta-analysis of more than 30 million births
Source: BMC Med. 2014 Nov 27;12:220. doi: 10.1186/s12916-014-0220-4 (PMC4245790; doi:10.1186/s12916-014-0220-4)
Supplement: Additional file 3: — Summary of subgroup analyses. Indented rows are subgroups of the higher categories. [file 12916_2014_220_MOESM3_ESM.docx]

**Table S1 - Summary of subgroup analyses**

Indented rows are subgroups of the higher categories.

| **Subgroups defined by:** | **Numbers of studies in contrasting groups** | **I^2^ for each subgroup** | **RRs for each subgroup (RE mode)** |
| --- | --- | --- | --- |
| Prenatal sex selection known  in country | 31 (No); 3 (Yes) | 69.3; 0% | 1.11; 0.64 |
| Gestational age definition of stillbirth^1^ | 20 (early, ≥20 weeks); 9 (late, ≥28 weeks)^*^ | 39.8%; 85.4% | 1.11; 1.12 |
| Population type^2^ | 2 (hospital ); 7 (population) | 33.6%; 88.7% | 1.12; 1.13 |
| Study design^2^ | 2 (case-control); 7 (cohort) | 5.8%; 88.8% | 1.11; 1.17 |
| Income group of country | 17 (High); 17 (Low-Middle) | 61.1%; 71.5% | 1.08; 1.10 |
| Raw vs. adjusted RRs used for meta-analysis | 8; 8 (same studies used for comparison) | 72.3%; 29.9% | 1.02, 1.07 |

2 studies defining stillbirth as death at ≥24 weeks, and 1 where time of death was undefined, were excluded from this analysis

^1^ In countries with no known prenatal sex selection

2 In countries with no known prenatal sex selection and late gestational age cut-off
